# Nebivolol reduces central blood pressure in stage I hypertensive patients: experimental single cohort study

**DOI:** 10.1590/1516-3180.2014.1325704

**Published:** 2014-07-22

**Authors:** Renan Oliveira Vaz-de-Melo, Luiz Tadeu Giollo-Júnior, Débora Dada Martinelli, Heitor Moreno-Júnior, Marco Antônio Mota-Gomes, José Paulo Cipullo, Juan Carlos Yugar-Toledo, José Fernando Vilela-Martin

**Affiliations:** I MD. Resident in Internal Medicine, Hospital de Base, Faculdade de Medicina de São José do Rio Preto (Famerp), São Paulo, Brazil; II BSc. Master's Student and Physiotherapist, Hospital de Base, Faculdade de Medicina de São José do Rio Preto (Famerp), São Paulo, Brazil; III BSc. Nurse, Hospital de Base, Faculdade de Medicina de São José do Rio Preto (Famerp), São Paulo, Brazil; IV MD, PhD. Full Professor, Department of Internal Medicine, Cardiovascular Pharmacology Laboratory, School of Medical Sciences, Universidade Estadual de Campinas (Unicamp), Campinas, São Paulo, Brazil; V MD. Full Professor, Universidade Estadual de Ciências Médicas de Alagoas (Uncisal), Maceió, Brazil; VI MD, PhD. Collaborating Professor, Department of Internal Medicine, Faculdade de Medicina de São José do Rio Preto (Famerp), São Paulo, Brazil; VII MD, PhD. Adjunct Professor, Head of Department of Internal Medicine and Coordinator of Hypertension Clinic, Faculdade de Medicina de São José do Rio Preto (Famerp), São Paulo, Brazil

**Keywords:** Adrenergic beta-antagonists, Vasodilator agents, Arterial pressure, Pulse wave analysis, Hypertension, Antagonistas adrenérgicos beta, Vasodilatadores, Pressão arterial, Análise de onda de pulso, Hipertensão

## Abstract

**CONTEXT AND OBJECTIVES:**

Assessment of central blood pressure (BP) has grown substantially over recent years because evidence has shown that central BP is more relevant to cardiovascular outcomes than peripheral BP. Thus, different classes of antihypertensive drugs have different effects on central BP despite similar reductions in brachial BP. The aim of this study was to investigate the effect of nebivolol, a β-blocker with vasodilator properties, on the biochemical and hemodynamic parameters of hypertensive patients.

**DESIGN AND SETTING:**

Experimental single cohort study conducted in the outpatient clinic of a university hospital.

**METHODS:**

Twenty-six patients were recruited. All of them underwent biochemical and hemodynamic evaluation (BP, heart rate (HR), central BP and augmentation index) before and after 3 months of using nebivolol.

**RESULTS:**

88.5% of the patients were male; their mean age was 49.7 ± 9.3 years and most of them were overweight (29.6 ± 3.1 kg/m^2)^ with large abdominal waist (102.1 ± 7.2 cm). There were significant decreases in peripheral systolic BP (P = 0.0020), diastolic BP (P = 0.0049), HR (P < 0.0001) and central BP (129.9 ± 12.3 versus 122.3 ± 10.3 mmHg; P = 0.0083) after treatment, in comparison with the baseline values. There was no statistical difference in the augmentation index or in the biochemical parameters, from before to after the treatment.

**CONCLUSIONS:**

Nebivolol use seems to be associated with significant reduction of central BP in stage I hypertensive patients, in addition to reductions in brachial systolic and diastolic BP.

## Abstract (pt)

**CONTEXTO E OBJETIVOS:**

A avaliação da pressão arterial central (PAc) tem crescido substancialmente nos últimos anos porque as evidências mostraram que PAc central é mais relevante para os desfechos cardiovasculares do que pressão arterial (PA) periférica. Assim, diferentes classes de anti-hipertensivos têm efeitos diferentes sobre PAc apesar de reduções semelhantes na PA braquial. O objetivo foi investigar o efeito do nebivolol, β-bloqueador com propriedades vasodilatadoras, nos parâmetros bioquímicos e hemodinâmicos de pacientes hipertensos.

**TIPO DE ESTUDO E LOCAL:**

Estudo de coorte única experimental realizado em ambulatório de hospital universitário.

**MÉTODOS:**

Todos os 26 pacientes recrutados foram submetidos à avaliação bioquímica e hemodinâmica (PA, frequência cardíaca, FC, PAc, augmentation index) antes e após três meses usando nebivolol.

**RESULTADOS:**

88,5% dos indivíduos eram do sexo masculino, com média de idade de 49,7 ± 9,3 anos, predominância de sobrepeso (29,6 ± 3,1 kg/m^2)^ e aumento da cintura abdominal (102,1 ± 7,2 cm). Houve diminuição significativa da PA sistólica periférica (P = 0,0020) e diastólica (P = 0,0049), da FC (P < 0,0001) e da PAc (129,9 ± 12,3 x 122,3 ± 10,3 mmHg, P = 0,0083) após o tratamento em comparação aos valores basais. Não houve diferença no augmentation index, nem nos parâmetros bioquímicos antes e após o período de tratamento.

**CONCLUSÕES:**

O uso de nebivolol parece estar associado à redução significativa da PAc em hipertensos estágio 1, além da redução da pressão sistólica e diastólica braquial.

## INTRODUCTION

Recent evidence has shown that central blood pressure (BP) is more relevant for predicting cardiovascular (CV) outcomes than peripheral pressure in the brachial artery.^1^ ^-^ ^4^ Since the publication of the Conduit Artery Function Evaluation (CAFE) study,1 the importance of assessments of arterial function and central blood pressure (BP) has increased substantially. Although brachial BP is a powerful predictor of CV morbidity and mortality,^3^ ^,^ ^4^ this measurement does not reflect the pressure in the central circulation.^5^ It has been shown that central BP is normally lower than peripheral BP, and many studies have shown a consistent relationship between central systolic BP and cardiovascular mortality. In addition, despite similar reductions in brachial BP, different classes of antihypertensive drugs have different effects on the central BP and arterial stiffness.6 Recent studies have shown that vasodilator antihypertensive drugs,^1^ ^,^ ^7^ ^,^ ^8^ such as renin-angiotensin system (RAS) inhibitors and calcium channel blockers, have a more favorable effect on indices of arterial stiffness than do older β-blockers, and particularly atenolol.^9^ ^,^ ^10^ It is conceivable that newer β-blockers with additional vasodilation properties may have favorable effects on arterial stiffness, compared with atenolol.

One such drug is nebivolol, a third-generation beta 1-selective β-blocker, which has favorable effects on carbohydrate and lipid metabolism, as well as on endothelial function and on oxidative stress. In recent studies, nebivolol was shown to improve artery stiffness to a greater extent than older β-blockers. It has been shown to have vasodilation properties in humans and animals.^11^ ^-^ ^14^ Among its properties are its ability to increase the bioavailability of nitric oxide (with consequently improvement of endothelial function), its antiproliferative effect and its ability to decrease oxidative stress.^15^ ^,^ ^16^ Because endothelial dysfunction and increased arterial stiffness play an important role in the early atherosclerotic processes and are associated with poor outcomes and increased mortality, independently of blood pressure, the ability of nebivolol to enhance the release of endothelium-derived nitric oxide, and consequently improve endothelial function and arterial stiffness,^7^ ^,^ ^8^ may have significant clinical implications for the use of this agent in treating hypertension and cardiovascular diseases. 

## OBJECTIVE

The aim of this study was to analyze the 12-week effect of nebivolol treatment on hemodynamic (BP, heart rate (HR), central BP and augmentation index) and biochemical parameters in stage I hypertensive patients without previous treatment.

## METHODS

### Study design, setting and sample

A total of 33 patients from our outpatient hypertension clinic who were interested in entering the study were initially screened for eligibility; among these, seven did not satisfy the inclusion/exclusion criteria. Thus, the sample for participation in this single-group prospective cohort study was composed of 26 stage I hypertensive patients. Stage I hypertension was defined as systolic BP ≥ 140 and < 160 mmHg and/or diastolic BP ≥ 90 and < 100 mmHg. Subjects presenting age < 18 years, hypertensive patients treated previously, obesity, alcohol abuse, current corticosteroid treatment, history of asthma, peripheral vascular disease, chronic kidney disease, secondary hypertension, unstable angina or previous myocardial infarction, previous stroke, heart failure, atrioventricular block, bradycardia < 50 bpm, pregnancy, uncontrolled diabetes mellitus or insulin therapy, neoplasia, history of drug abuse or any other clinical conditions associated with poor prognosis were excluded.

Eligible participants visited our clinical research laboratory at 7:00 am after a 12-hour fast, to undertake the protocol procedures. Anthropometric variables were measured and blood was sampled for the lipid profile and routine laboratory parameters (hemoglobin, hematocrit, platelets, leukocytes, glutamic oxaloacetic and pyruvic transaminases, serum creatinine, glucose, urea and total bilirubin). LDL-cholesterol was calculated using the Friedewald formula.^17^ Furthermore, all patients underwent BP recordings and determination of central aortic BP in the consultation office, and determination of the augmentation index by means of applanation tonometry on peripheral arteries, as described below. All the latter measurements were made in a quiet room with controlled air temperature (approximately 22 ºC). 

Treatment with nebivolol started after confirmation of the inclusion criteria. The patients received nebivolol at a dose of 5 mg/day. Follow-up visits for BP measurements, physical examination and study medication dispensation were made every month. The study participants' adherence to the therapy administered was assessed at the follow-up visits by means of tablet counts. At the end of the study (three months), the baseline measurements were repeated for all of the 26 patients evaluated initially. 

### Assessments

Peripheral BP at the level of the brachial artery was measured in the seated position after a ten-minute rest, using a mercury sphygmomanometer and cuffs with bladder size encircling at least 80% of the upper-arm circumference and covering two-thirds of the upper-arm length.18 Brachial BP was measured in both arms, and if there was a difference in BP levels between the two arms, the measurements in the arm with the higher BP were taken into account. Three BP measurements with at least a one-minute interval between them were obtained, and the mean of the three measurements was recorded. Phase I and V Korotkoff sounds were recorded for systolic BP (SBP) and diastolic BP (DBP), respectively.

### Pulse-wave analysis

*Central blood pressure and augmentation index*

Arterial pulse waveforms from the left radial artery were measured noninvasively by means of an automated tonometry system (HEM- 9000 AI; Omron Healthcare Co. Ltd., Kyoto, Japan), after the participants had rested in a seated position for 10 minutes. Pulsewave analyses were performed at least three times and the mean of the measurements was calculated. The radial arterial waveforms from this device were used to calculate the augmentation index (AIx). The first systolic peak (SBP1) and the late (second) systolic peak (SBP2) were automatically identified using the fourth-derivative wave as the second and third zero crossing points, respectively. The augmentation index (AIx) was defined as the ratio of the height of SBP2 to that of SBP1. The brachial BP and heart rate (HR) were measured simultaneously in the right brachial artery using an oscillometric device incorporated in the HEM-9000 AI device. Late systolic BP in the radial artery, as an index of central BP, was calculated using the following equation: rSBP2 = r-AIx. (brachial systolic BP - brachial diastolic BP) + brachial diastolic BP. The HR-adjusted augmentation index (AIx(75)) was calculated by adjusting AIx at an inverse rate of 4.8% for each ten-beats-perminute increment in heart rate. All measurements were performed by a single examiner, after the subject had fasted for at least eight hours, both before and after three months of nebivolol use.^19^ ^,^ ^20^

### Sample-size calculation

A power analysis calculation was conducted using the site http://www.lee.dante.br/pesquisa/amostragem/calculo_ amostra.html. Assuming an error of 0.01 and a study power of 80%, the calculated size of the sample required in order to reject the hypothesis of nullity was 23.

### Statistical analysis

Descriptive analysis was performed for qualitative variables, and quantitative results are presented as means ± standard deviations. The Wilcoxon test was used to compare quantitative variables before and after treatment with nebivolol, among these stage I hypertensive patients without previous treatment. All statistical analyses were performed using the Minitab 15.0 statistics software. For all tests, a P-value < 0.05 was considered significant.

### Ethical authorization

This study was approved by the Research Ethics Committee of the Medical School and was registered under no. 312/2008. The study protocol was approved by the National Ethics Committee (CONEP-372/2007). Informed consent was obtained from all participants, in relation to both the treatment and the biochemical and hemodynamic evaluation.

## RESULTS

The sample was composed of 26 patients (88.5% male) with a mean age of 49.7 ± 9.3 years, who were predominantly overweight (29.6 ± 3.1 kg/m^2)^ and had large abdominal waist circumference (102.1 ± 7.2 cm). Significant decreases in systolic BP (P-value = 0.0020) and diastolic BP (P-value = 0.0049) were observed during the treatment with nebivolol, and these were associated with decreases in heart rate (P-value < 0.0001) and central BP (P-value = 0.0083) (Table 1). There was no difference in the AIx, with or without correction for heart rate, during the treatment period.

**Table 1:** Comparison of hemodynamic (peripheral and central) and biochemical parameters before and after treatment with nebivolol among stage I hypertensive patients

Variable	Initial	After treatment	P-value
*Peripheral hemodynamic parameters*			
Brachial systolic blood pressure (mmHg)	137.9 ± 11.5	128.5 ± 7.7	0.0020
Brachial diastolic blood pressure (mmHg)	85.5 ± 9.7	78.0 ± 9.0	0.0049
Brachial pulse pressure (mmHg)	52.4 ± 9.6	50.5 ± 9.0	NS
Heart rate (bpm)	74.1 ± 8.4	64.0 ± 8.3	< 0.0001
*Central tonometry parameters*			
Augmentation index (%)	86.7 ± 9.8	89.9 ± 11.7	NS
Augmentation index corrected for heart rate (%)	87.0 ± 8.5	85.4 ± 10.0	NS
rSBP2 (mmHg)	129.9 ± 12.3	122.3 ± 10.3	0.0083
*Biochemical parameters*			
Hemoglobin (mg/dl)	15.8 ± 1.4	15.6 ± 0.9	NS
Hematocrit (%)	46.8 ± 3.6	46.6 ± 2.7	NS
Platelets (x10^3^)	227.4 ± 40.4	216.4 ± 36.4	NS
Leukocytes (x10^3^)	7.1 ± 1.4	7.0 ± 1.5	NS
Glutamic oxaloacetic transaminase (U/l)	26.2 ± 10.7	22.6 ± 6.1	NS
Glutamic pyruvic transaminase (U/l)	40.8 ± 33.5	33.7 ± 14.9	NS
Creatinine (mg/dl)	1.2 ± 0.1	1.1 ± 0.2	NS
Glucose (mg/dl)	96.3 ± 14.1	94.5 ± 12.0	NS
Potassium (mEq/l)	4.3 ± 0.4	4.2 ± 0.4	NS
Total cholesterol (mg/dl)	237.3 ± 49.1	222.8 ± 39.1	NS
HDL-cholesterol (mg/dl)	59.3 ± 25.7	56.5 ± 39.1	NS
LDL-cholesterol (mg/dl)	129.1 ± 31.5	121.7 ± 29.3	NS
Triglycerides (mg/dl)	244.4 ± 232.1	219.7 ± 128.3	NS
Urea (mg/dl)	35.5 ± 9.1	40.0 ± 14.5	NS
Total bilirubin (mg/dl)	0.9 ± 0.5	0.8 ± 0.3	NS

**rSBP2:** = Late systolic blood pressure in the radial artery (= central blood pressure)

NS = non-significant (P > 0.05).

**Figure 1:**
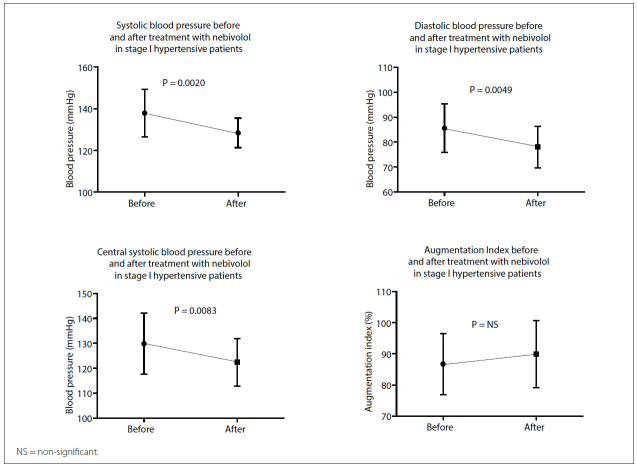
Comparison of systolic blood pressure, diastolic blood pressure, central systolic blood pressure and augmentation index before and after treatment with nebivolol in stage I hypertensive patients.

Table 1 displays the routine biochemical characteristics and the pulse pressure, heart rate, SBP and DBP levels at brachial artery level and tonometry parameters (AIx, AIx(75) and central BP). Body mass index, abdominal waist measurement, fasting plasma glucose, serum lipids and other biochemical parameters did not differ between before and after treatment. Figure 1 shows a comparison of hemodynamic parameters (peripheral and central) between the basal and the follow-up evaluations among the patients treated with nebivolol. In this study, nebivolol significantly reduced brachial BP and central BP, even though the AIx and biochemical parameters remaining unchanged. 

## DISCUSSION

In the present study, we examined the 12-week effect of nebivolol on biochemical and tonometry parameters among stage I hypertensive patients without previous treatment. To our knowledge, this is the first study on Brazilian individuals to evaluate the beneficial effects of nebivolol on the hemodynamic profile, and it showed worthwhile reductions in peripheral and central blood pressure.

This study investigated in detail the potential effects of nebivolol, a beta-1 selective beta-blocker, on aortic stiffness, wave reflections and central hemodynamic parameters in patients with mild hypertension that had never been treated. In this study, nebivolol significantly reduced brachial and central BP, even though the AIx remained unchanged. 

Brachial BP measurement is considered to be the best method for screening for and diagnosing clinical hypertension. However, over recent years, assessment of CV risk in subjects with hypertension has led to development of more sophisticated methods of BP measurement, such as central BP.^21^ Currently, several arguments suggest that central BP is more relevant than peripheral (brachial) BP for determining CV risk assessments.^19^ ^,^ ^21^ ^,^ ^22^ The pressure wave generated by the left ventricle travels down the arterial tree and then is reflected at any discontinuity of the arterial wall, especially multiple-resistance arterioles and their bifurcations. Thus, the pressure waveform recorded at any site of the arterial tree is the sum of a forward travelling waveform generated by left ventricular ejection and a backward travelling wave, reflected at peripheral sites.^21^ ^,^ ^22^ As a consequence of transmission of the pressure wave and reflections, SBP and pulse pressure (PP) are amplified by as much as 10-15 mmHg when moving from the aorta to the brachial artery. This phenomenon, which is not taken into consideration in published clinical guidelines, has three major consequences, which relate to CV complications of hypertension, the choice of antihypertensive drugs and HR regulation. 

Regarding CV complications, when large-conduit arteries are healthy and compliant (young individuals), the reflected wave merges with the incident wave in the proximal aorta mostly during diastole, thereby augmenting aortic DBP and supporting coronary perfusion. In contrast, when the arteries are stiff (old individuals), wave travel is faster and the reflected wave merges earlier with the incident wave, thus augmenting aortic systolic rather than diastolic pressure. As a result, left ventricular afterload is increased and coronary filling is compromised. This pathophysiological mechanism supports the idea that central BP is superior to peripheral BP for predicting CV risk and that it acts on CV risk independently of atherosclerosis and other traditional CV risk factors.^19^ ^,^ ^21^ ^,^ ^22^

The choice of drug treatment for hypertension is also influenced by wave reflections: both by their amplitude (i.e. the proportion of the incident wave which is reflected) and by their timing. Acutely, vasodilator drugs reduce the amplitude of wave reflections and hence SBP.^21^ This situation is observed typically with nitrates, but may also occur with SRA inhibitors or calcium channel blockers. With the presence of chronic hypertension, arterial and arteriolar remodeling modifies the baseline characteristics (geometry, distensibility and structure) of reflection sites, particularly at the arteriolar level. Under drug treatment, central SBP will be consistently reduced if vascular remodeling and reflection coefficients are adequately corrected (by means of SRA or calcium channel blockade), but will remain elevated if the structures of the microvasculature and the reflection coefficients remain poorly modified despite drug treatment (β-blockers).^21^

It is well accepted that calcium channel blockers and drugs acting on the RAS improve endothelial function and arterial stiffness.^22^ ^-^ ^24^ On the contrary, β-blockers have failed to show positive effects on vascular function and central hemodynamic parameters.^1^ ^,^ ^25^ ^,^ ^26^ For this reason, recent studies have questioned whether β-blockers are still an appropriate therapy for uncomplicated hypertension.^1^ ^,^ ^25^ ^,^ ^27^ Since β-blockers are a heterogeneous class of drugs with different pharmacological and physiological properties, it may not be possible to extrapolate the results gathered from these studies using atenolol, to other drugs of the same class. It has been well demonstrated that regardless of similar reductions in brachial BP, there are several differences between the currently available β-blockers. 

Our results, along with other recent findings, support the conclusion that nebivolol has vascular effects that are more favorable than those of first and second-generation β-blockers. In a double-blind randomized study comparing nebivolol and metoprolol, Kampus et al.^8^ demonstrated that there was a significant reduction in brachial BP with a significant reduction in central BP only in the nebivolol group. In untreated hypertensive patients randomized to receive nebivolol or atenolol, Mahmud et al. observed a significant reduction in the brachial BP and pulse wave velocity associated with a significant reduction in AIx in the nebivolol group.^7^ Polónia et al. showed that for similar brachial BP and aortic stiffness, treatment with nebivolol was associated with lower central systolic BP than treatment with atenolol.^6^ On the other hand, Vitale et al. showed that nebivolol was not inferior to the angiotensin-receptor blocker irbesartan for improving endothelial function, arterial stiffness and central hemodynamic parameters in uncomplicated hypertensive patients.^28^

Studies on Caucasian and African-American subjects have suggested that the less effective central blood pressure control and consequently lower cardiovascular protection with older β-blockers may be due to an adverse effect from heart rate lowering on arterial wave reflection.^29^ Because of the vasodilator effect, the reduction in heart rate with nebivolol is lower than with other β-blockers, which leads to decreased wave reflection and improvement in arterial stiffness.^30^ Peripheral vasodilation also may contribute towards reducing the cardiac afterload, and towards reverting adverse arterial remodeling. Moreover, arterial stiffness is linked to endothelial dysfunction and reduced bioavailability of nitric oxide,^11^ a phenomenon that can be reduced with drugs that increase nitric oxide production, such as nebivolol. Our findings would support this mechanism and would explain the decrease in central aortic pressure observed in this study.

The present study had several limitations. These included the duration of follow-up, lack of a control group and the study design. If the patients had been followed for a longer time, perhaps we would have observed changes in the AIx. The lack of a comparison group, in our viewpoint, was the main limitation of this study. The randomised, placebo-controlled, and double blind trial represents the gold standard in evidence based medicine. However, this does not invalidate the significant results of central BP reduction after 12 weeks of treatment with nebivolol. Although brachial BP remains the principal tool used for the clinical diagnosis and monitoring of hypertension, there is an increasing body of evidence demonstrating that central BP measurement may be a better prognostic marker for hypertension. 

Moreover, recent evidence suggests that some antihypertensive drugs can influence central BP more consistently than peripheral BP. This is especially true for agents acting on the RAS or calcium channel blockade, as well as newer β-blockers. Nevertheless, large prospective studies aiming to compare the predictive value of peripheral and central BP in the general population, as well as studies comparing the effectiveness of hypertension management based on peripheral BP measurements, compared with central BP measurements, are needed before algorithms based on central BP can be recommended for clinical practice.

## CONCLUSIONS

In summary, nebivolol presents a favorable effect as an antihypertensive drug with possible additional capacity to improve arterial stiffness by reducing central BP, a characteristic not exhibited by other β-blockers.
